# Identification and characterization of circular RNAs expression profiles in obstructive sleep apnea-induced liver injury

**DOI:** 10.18632/aging.205701

**Published:** 2024-03-21

**Authors:** Chaowei Li, Jinhuang Lin, Qingshi Chen, Yueyong Zhu

**Affiliations:** 1 Department of Hepatology, Hepatology Research Institute, The First Affiliated Hospital of Fujian Medical University, Fuzhou 350001, China; 2 Department of Gastroenterology, The Second Affiliated Hospital of Fujian Medical University, Quanzhou, China; 3 Department of Neurointervention, Zhangzhou Affiliated Hospital of Fujian Medical University, Zhangzhou, China; 4 Department of Endocrinology, The Second Affiliated Hospital of Fujian Medical University, Quanzhou, China; 5 Department of Hepatology, Fujian Clinical Research Center for Liver and Intestinal Diseases, Fuzhou 350001, China

**Keywords:** obstructive sleep apnea (OSA), chronic intermittent hypoxia (CIH), liver injury, circular RNAs (circRNAs)

## Abstract

Circular RNAs (circRNAs) have exhibited microRNA sponge activity, related to many important biological processes. Our study attempted to explore the comprehensive changes of circRNAs expression pattern in Obstructive sleep apnea (OSA)-induced liver injury and provide a global perspective of differentially expressed circRNAs (DECs). Then, RT-qPCR was used to confirm the microarray data. Further, gene ontology (GO) and KEGG pathway analysis were performed to annotate the DECs. Finally, the circRNA-miRNA-mRNA interaction network was established to predicted the target genes and target miRNAs of DECs for a stepwise bioinformatics analysis. We revealed a total of eighty DECs. In the meantime, six circRNAs were randomly validated by RT-qPCR. Among these circRNAs, mmu_circRNA_000469, 37851, 38959, 38983, 31665 were up-regulated in both microarray and qRT-PCR tissues, while mmu_circRNA_27565 was down-regulated. GO analysis revealed that circRNAs-target genes were largely related to liver function process such as carboxylic acid metabolic process and negative regulation of mitochondrial membrane potential. Meanwhile, KEGG analysis found that there were 13 pathways related to these circRNAs- target genes. And the most enriched pathway was Natural killer cell mediated cytotoxicity, which strongly suggests that immune responses may be important for the process of OSA-induced liver injury. In addition, four significant DECs (mmu_circRNA_000469, 38959, 38983, 27565) and their target mRNA and target miRNAs were further selected to establish the regulation network. Our study revealed that circRNAs may play a crucial role in OSA-induced liver injury and thus mmu_circRNA_000469, 38959, 38983, 27565 may serve as biomarkers of biological process of OSA-induced liver injury.

## INTRODUCTION

Obstructive sleep apnea (OSA) is a sleep-related breathing disorder leading to chronic intermittent hypoxia (CIH) during sleep, affecting 9~15% of middle-aged adults worldwide [1]. OSA is a common condition with major cardiovascular consequences. At present, a great deal is known about the role of OSA in cardiovascular diseases such as myocardial infraction [2] and cardiac injury [3]. Previous studies show that severe OSA is a risk factor for abnormal liver enzymes and steatohepatitis independent of overweight states [4]. Promotion of insulin resistance and systemic inflammation were possible contributing factors in the process of OSA-induced liver injury [5]. However, the knowledge of potential mechanism underlying the pathogenesis of OSA-induced liver injury is thus very limited. Therefore, it is urgently needed to better understand the pathogenesis of OSA-associated liver injury, which may greatly improve its diagnosis and treatment.

As a subclass of non-coding RNAs, circular RNAs (circRNAs) are characterized by joining the head 3′ of the RNA to its tail 5′ ends, resulting in a circular form. CircRNAs interact with microRNA (miRNA) and function as miRNA sponges [6]. Recent studies have unveiled that circRNAs play an important role in liver diseases [7, 8]. This mechanism involves ceRNA (miRNA sponges), relating to different liver diseases [7, 9].

Recently, Zhao et al. found that steatohepatitis-associated circRNA ATP5B Regulator (SCAR) constrains mitochondrial ROS output and stimulates fibroblast, thus suppressing the process from NASH to liver fibrosis [9]. However, the expression profile and potential role of circRNAs in OSA-induced liver injury has not been fully explored so far. Thus, we hypothesized that the mutation of circRNAs may be closely related to the pathogenesis of OSA-induced liver injury. In this research, we explored the differentially expressed circRNA (DECs) in a CIH model to identify potential circRNAs associated with liver injury. Specifically, for identifying the DECs, we used the circRNAs microarray screening, confirmed by quantitative real-time PCR (qRT-PCR) and performed a stepwise bioinformatics analysis. Our findings may bring a novel perspective towards a better understanding of OSA-induced liver injury.

## MATERIALS AND METHODS

### Animals

Six-week-old male balb/c mice (17–21 g) were purchased from Beijing Weitong Lihua Experimental Animal Technology Co., Ltd. All mice were housed with standard mouse diet and tap water. The animal protocol of our study was approved by the Experimental Animal Ethics Committee of the Second Affiliated Hospital of Fujian Medical University (QZ5312408).

### Liver CIH injury protocol and circRNAs sequencing

An CIH model was established by chronic intermittent hypoxia (IH) as previously described by us [3, 10]. Briefly, we used the intermittent hypoxia system to create a CIH environment. Specifically, the oxygen and nitrogen were automatically flowed into the chamber by a gas control system and calculated with an oxygen analyzer, creating an intermittent hypoxia condition. After the chamber reached 6% O_2_ for 1 min with a 2 cycle (1 min/cycle), compressed air was pumped into the chamber to achieve 21% O_2_ for another 1 min. Six mice were randomly allocated into two groups (*n* = 3, each group): (1) an CIH group, in which mice were placed daily in the chamber for 30 cycles/h, 8 h/day for 7 days/week, for eight consecutive weeks; (2) a control group, in which mice were housed in the chamber with 21% O_2_ during the entire experiment. Following pentobarbital euthanasia, the liver tissues of mice were collected at the end of CIH exposure. All mice experiments were performed in duplicate and repeated at least three times.

### CircRNAs microarray hybridization and circRNAs microarray analysis

**Figure 1:**
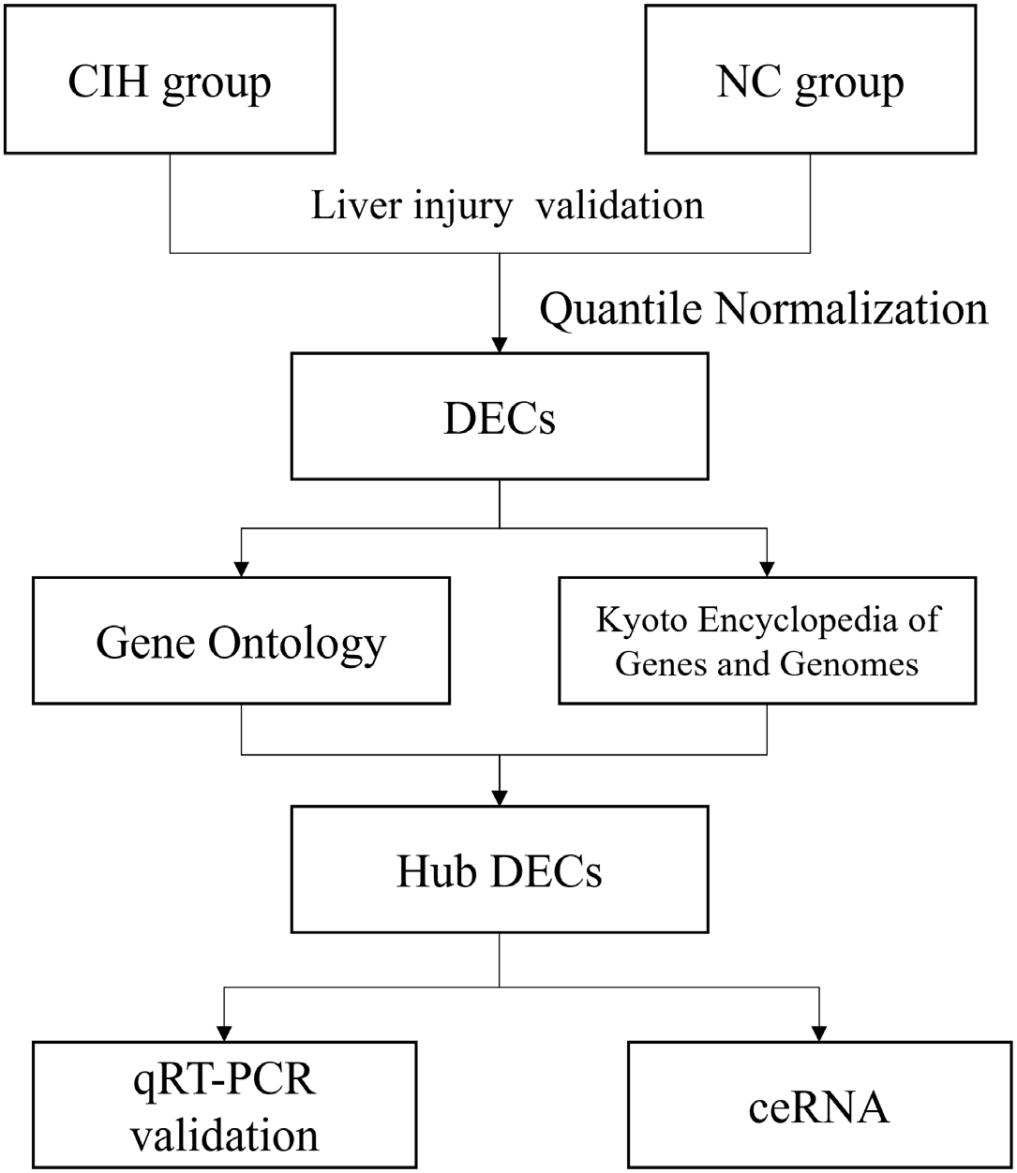
The flowchart in this study.

The general flowchart of data processing and detailed methods were described in Figure 1. Briefly, the process detail of circRNA microarray hybridization can be found in our previous work [3, 7]. Agilent Microarray Scanner was used to scan the arrays and acquired array images analyzed using Agilent Feature Extraction Software 9.5.1.1. Quantile normalization and subsequent data processing were performed using R project and the Bioconductor package of Limma [11, 12].

Changes in circRNAs expression with *p*-values < 0.05 and fold-changes ≥1.5 were considered statistically significant. Fold change filtering was calculated for screening DECs, which were visualized with Volcano Plot. Hierarchical clustering was performed to display the distinguishable DECs patterns between samples.

### Bioinformatic analyses

Gene Ontology (GO) enrichment analysis was performed to determine functional annotations of DECs-target genes, including biological processes, cellular components and molecular functions, using the clusterProfiler R package (v4.0.0) [13, 14]. Furthermore, Kyoto Encyclopedia of Genes and Genomes (KEGG) analysis was also further performed to identify the enriched pathways among these DECs-target genes (Kanehisa and Goto, 2000). The threshold of significance was defined as *P* ≤ 0.05 for both GO and KEGG analyses. Moreover, we ranked the top 10 enriched GO categories and the top 10 enriched KEGG pathways of the DECs-target genes.

### Validation by qRT-PCR

We used qRT-PCR to validate the differential expression level of six selected circRNAs. The total RNA from liver tissues was extracted by using the TRIzol Reagent (Takara, Dalian, China). All primers, spanning the distal ends of circRNAs, were designed using Primer 5 software (Table 1). Briefly, cDNA was synthesized using the PrimeScript™ RT Reagent Kit (Takara, China). Subsequently, the qRT-PCRs were run and analyzed on an ABI Q2Real-time PCR system (Applied Biosystems, USA) using a TB Green™ Premix Ex Taq™ II (Takara, China). Finally, relative circRNAs expression were calculated using the 2^−ΔΔCt^ method, with β-actin serving as an internal control.

**Table 1:** Primers used for qRT-PCR.

**Genes**	**Forward and reverse sequence**	**Product length (bp)**
ACTB (Internal Control)	F:5′-GTACCACCATGTACCCAGGC-3′	247
	R:5′-AACGCAGCTCAGTAACAGTCC-3′	
mmu_circRNA_000469	F:5′-GCAGAAGAAGGCAAAAAAAGGT-3′	135
	R:5′-AGTGGGTTTATCAGGCAATCG-3′	
mmu_circRNA_37851	F:5′-GGGGAGGTGAATCGGTTTTC-3′	204
	R:5′-GGATCTTTGTGTCCAGGTCTGTC-3′	
mmu_circRNA_38983	F:5′-GGAGTGGAATGGAAAAACGG-3′	92
	R:5′-ATGCCTTATTGGTGACAGCAGA-3′	
mmu_circRNA_38959	F:5′-GGAGAAGCAGATTAAGAAACAAACC-3′	79
	R:5′-CACGAGAGTTGGGGTTGACAC-3′	
mmu_circRNA_31665	F:5′-TCAGAAGTGGACCTGCCGAC-3′	114
	R:5′-GAGAGCCCAAGGGATTTCATAA-3′	
mmu_circRNA_27565	F:5′-ATCTTGTTGTATGCCCTGACCT-3′	269
	R:5′-GGAAGTCCAAATGTGTCCAGAG-3′	

### Construction of circRNA-miRNA-mRNA networks

Based on the database of miRanda and TargetScan, we randomly selected four significant DECs to construct a competitive network among circRNA, miRNA and mRNA by a software (Arraystar’s home-made miRNA target prediction software). In addition, the binding capacity of circRNA and microRNA, as well as the capacity and number of microRNA-mRNA binding sites should be considered when we used the software. Finally, we estimated a circRNA-miRNA-mRNA network to analyze the ceRNA manner of the circRNAs and the network was visualized with Cytoscape software (Version 3.7.2) [15].

### Statistical analysis

Each measurement was independently repeated three times. Continuous data were expressed as mean ± standard deviation (SD). All qPCR statistical analyses using unpaired student’s *t*-test. *P* < 0.05 were considered marginally significant.

## RESULTS

### CIH treatment affected liver histology

**Figure 2:**
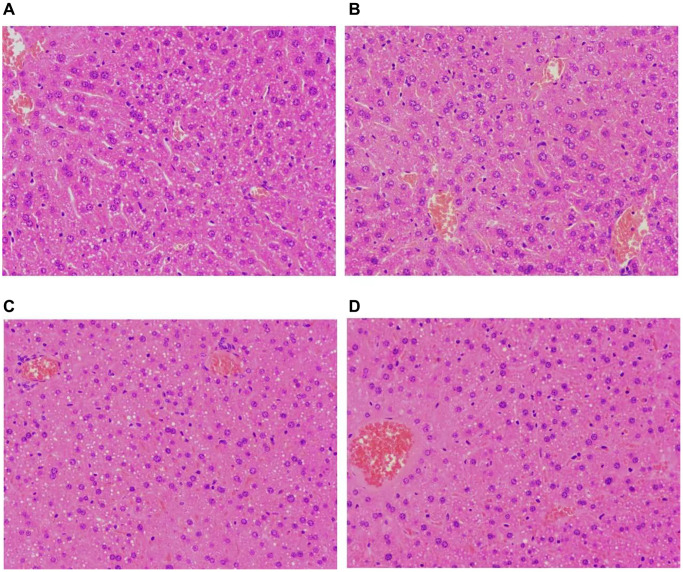
**Histopathological alterations involving liver tissues.** (**A**, **B**) Representative images of HE-stained liver tissues of the NC group; (**C**, **D**) Representative images of HE-stained liver tissues of the CIH group. NC group had normal hepatic architecture; CIH group showed some liver cells were slightly edematous with unclear borders. A few hepatocyte nuclei were slightly enlarged. Some cells were in sheets, and the demarcation of the hepatic cord was unclear. All images H&E with original magnification of 200X. NC, normal control; CIH, chronic intermittent hypoxia.

Normal hepatic architecture was observed in the liver tissues of NC group (Figure 2A, 2B). As shown in Figure 2C, 2D, CIH caused liver injury evidenced by some liver cells slightly edematous with unclear borders and a few hepatocyte nuclei slightly enlarged infiltrated (Figure 2C, 2D).

### Overview of circRNAs expression

**Figure 3:**
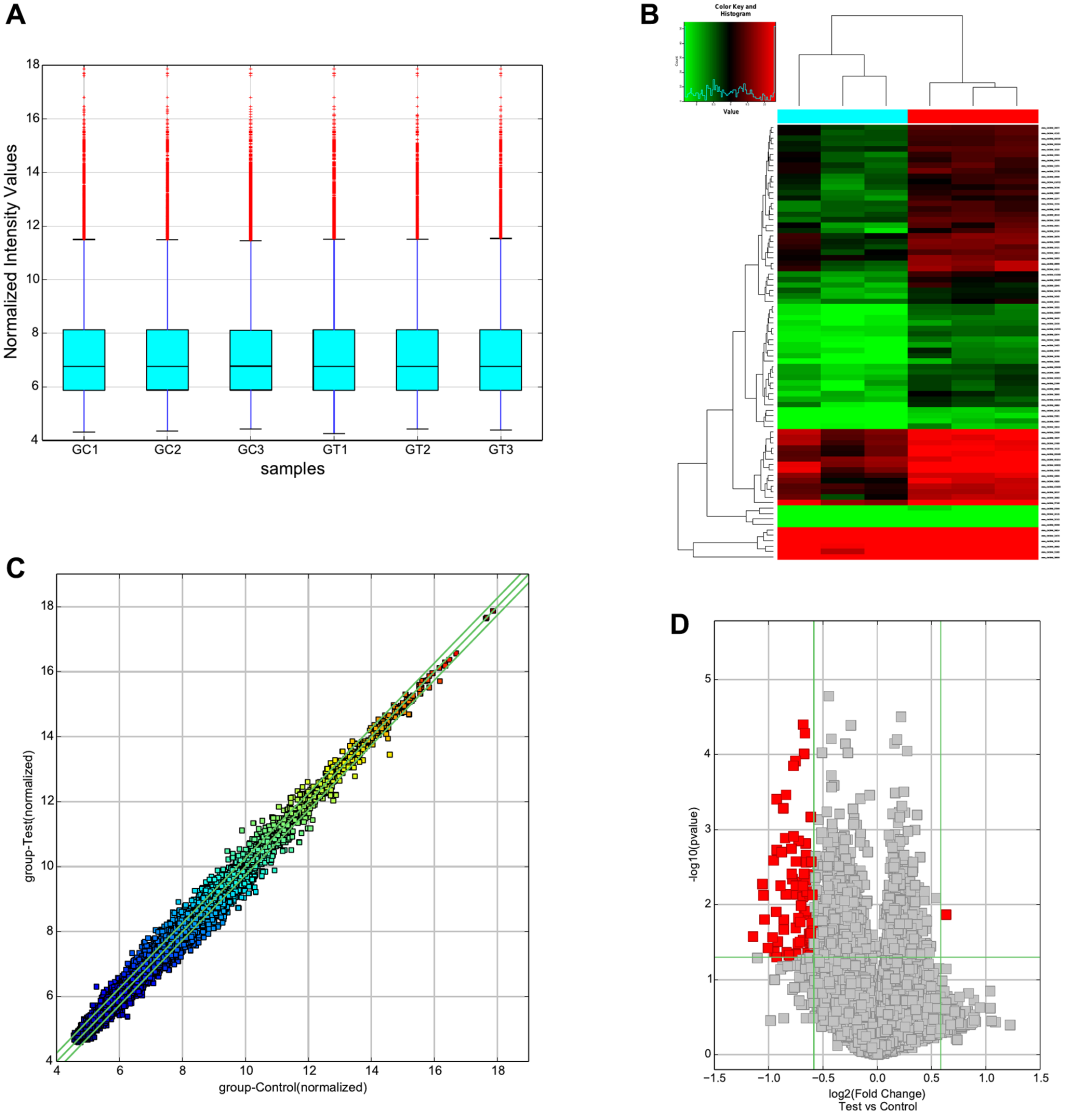
**Overview of the microarray signatures.** (**A**) Box plots show the normalized intensities from the two groups. (**B**) Hierarchical clustering of all DECs with a fold-change ≥1.5 and *p*-value < 0.05. (**C**) CircRNAs in the Scatter-Plot located above the top green line and below the bottom green line indicated more than a 1.5-fold change of circRNAs. (**D**) Volcano plot filtering revealed that 80 DECs, including 1 up-regulated and 79 down-regulated circRNAs. Abbreviation: DECs: differentially expressed circRNAs.

**Figure 4:**
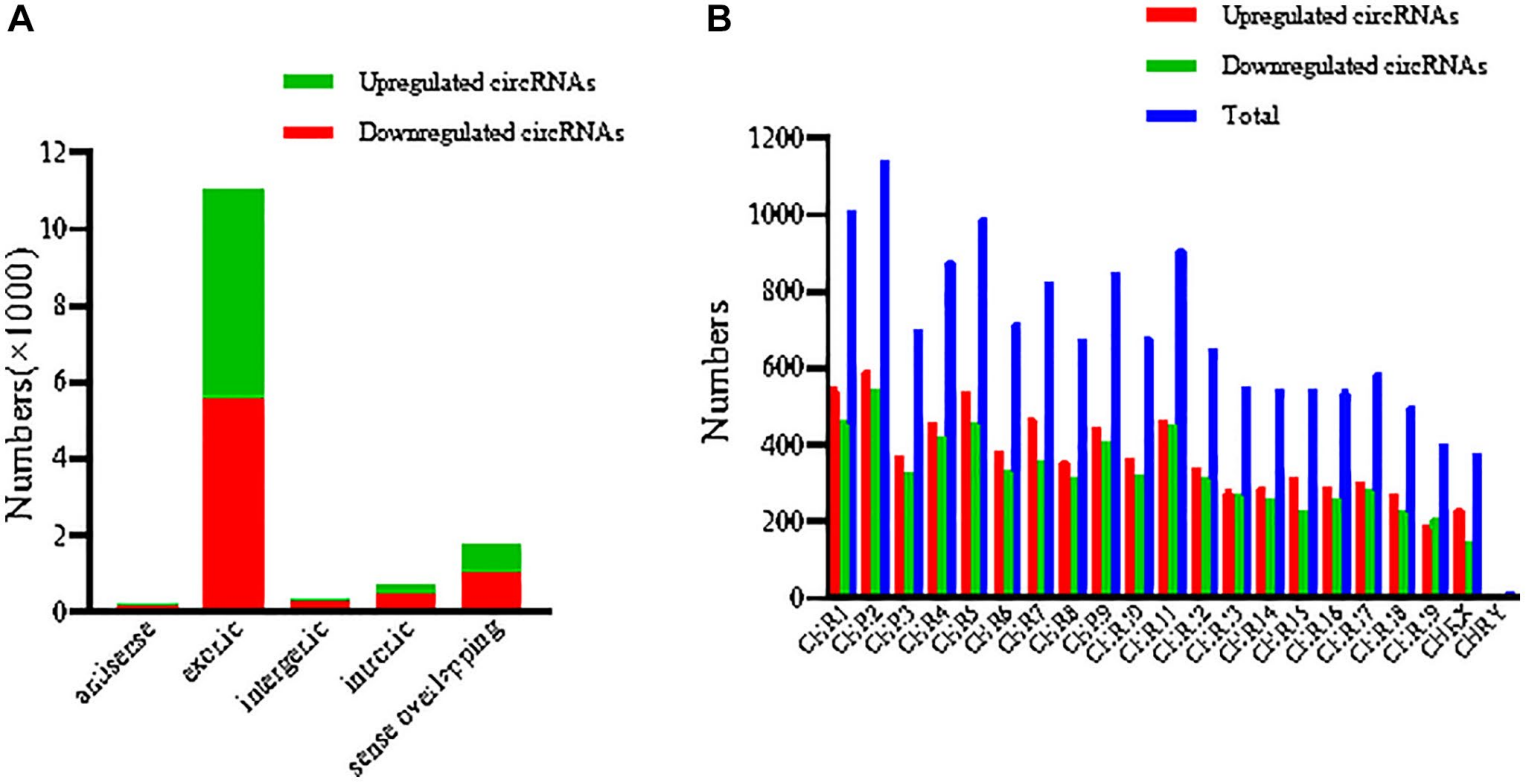
**Classification and distribution of the DECs.** (**A**) Classification of the DECs was listed. (**B**) The number of DECs was determined in each chromosome. DECs, differentially expressed circRNAs.

CircRNA microarray was conducted to screen the dysregulated circRNAs from 3 CIH-liver tissues and 3 normal liver tissues from the control group. With the above *p*-value and fold-change threshold, we detected a total of 80 DECs. Of these DECs, 1 were upregulated, while 79 were downregulated. The results of the quantile regression in Figure 3A show that the circRNA median of the six samples were normalized by quantile normalization (Figure 3A). Importantly, hierarchical clustering, scatter plots and a volcano plot efficiently show that clearly DECs expression profiles in two groups (Figure 3B–3D). To classify the dysregulated circRNAs in CIH-liver tissue samples, we listed the circRNAs into five categories, including 5567 exonic, 1026 sense-overlapping, 430 intronic, 240 intergenic regions and 140 antisense of the 7403 upregulated circRNAs, while the 6588 downregulated circRNAs comprised 5456 exonic, 733 sense-overlapping, 258 intronic, 74 intergenic and 66 antisense (Figure 4A). In addition, chromosomal distribution analysis revealed that most circRNAs were located at chromosome 1,2,4,5,9,11, while few located at the Y chromosome (Figure 4B).

### Validation of circRNA expression

**Figure 5:**
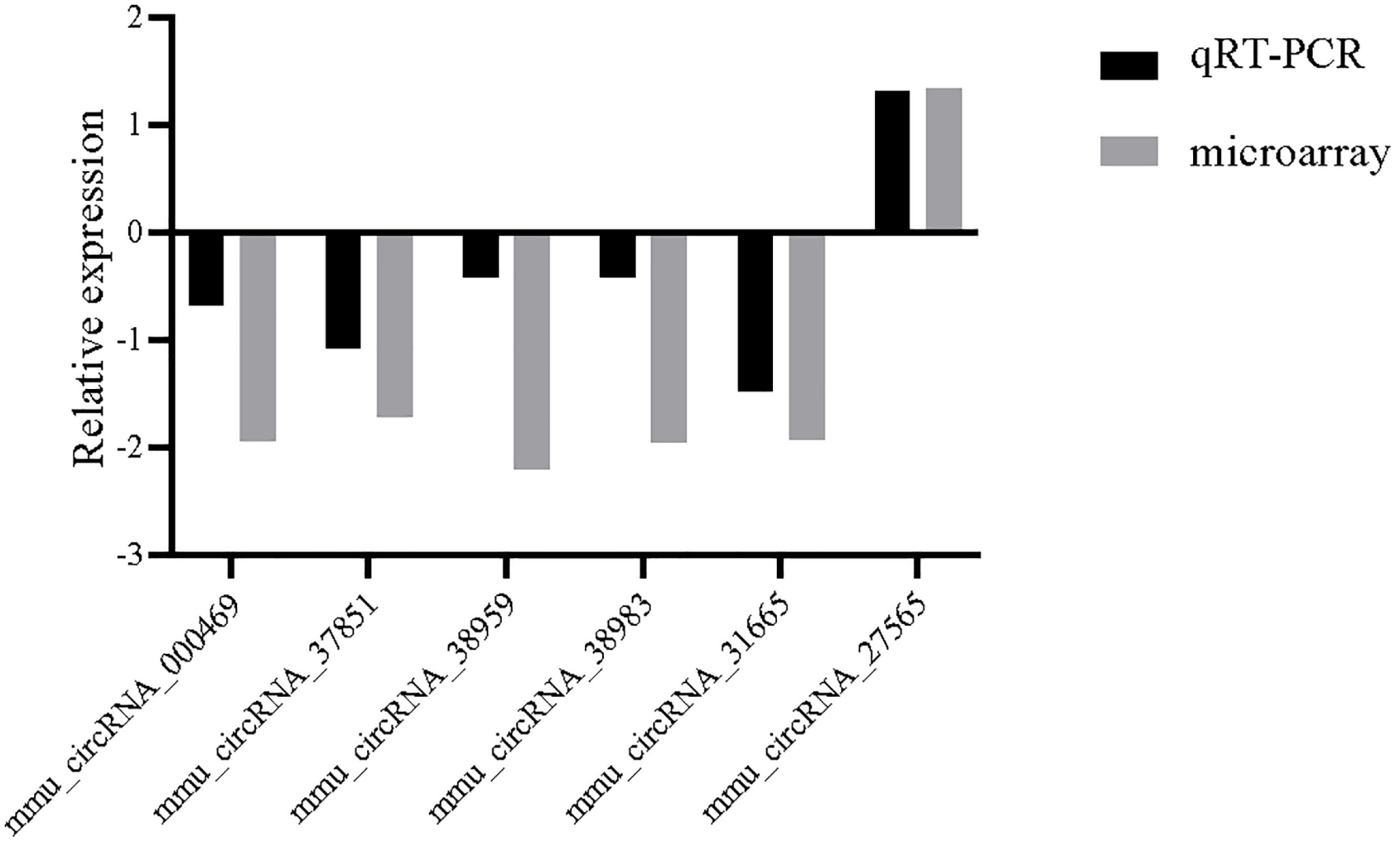
**Relative fold changes of six randomly selected circRNAs from microarray results and validated by qRT-PCR.** The downward and upward histogram represent down-expression and up-expression, respectively.

To verify the veracity of microarray results, we conclusively validated the selected circRNAs by qRT-PCR. There are 5 downregulated circRNAs (mmu_circRNA_000469, 37851, 38959, 38983, 31665) and 1 upregulated circRNAs (mmu_circRNA_27565) between the CIH group and the control group. These six circRNAs are selected according to a comprehensive evaluation of the *p*-values, fold-changes, length and original signal values of the circRNA. The qRT-PCR data were in agreement with the above microarray results (Figure 5).

### GO and KEGG analyses

**Figure 6:**
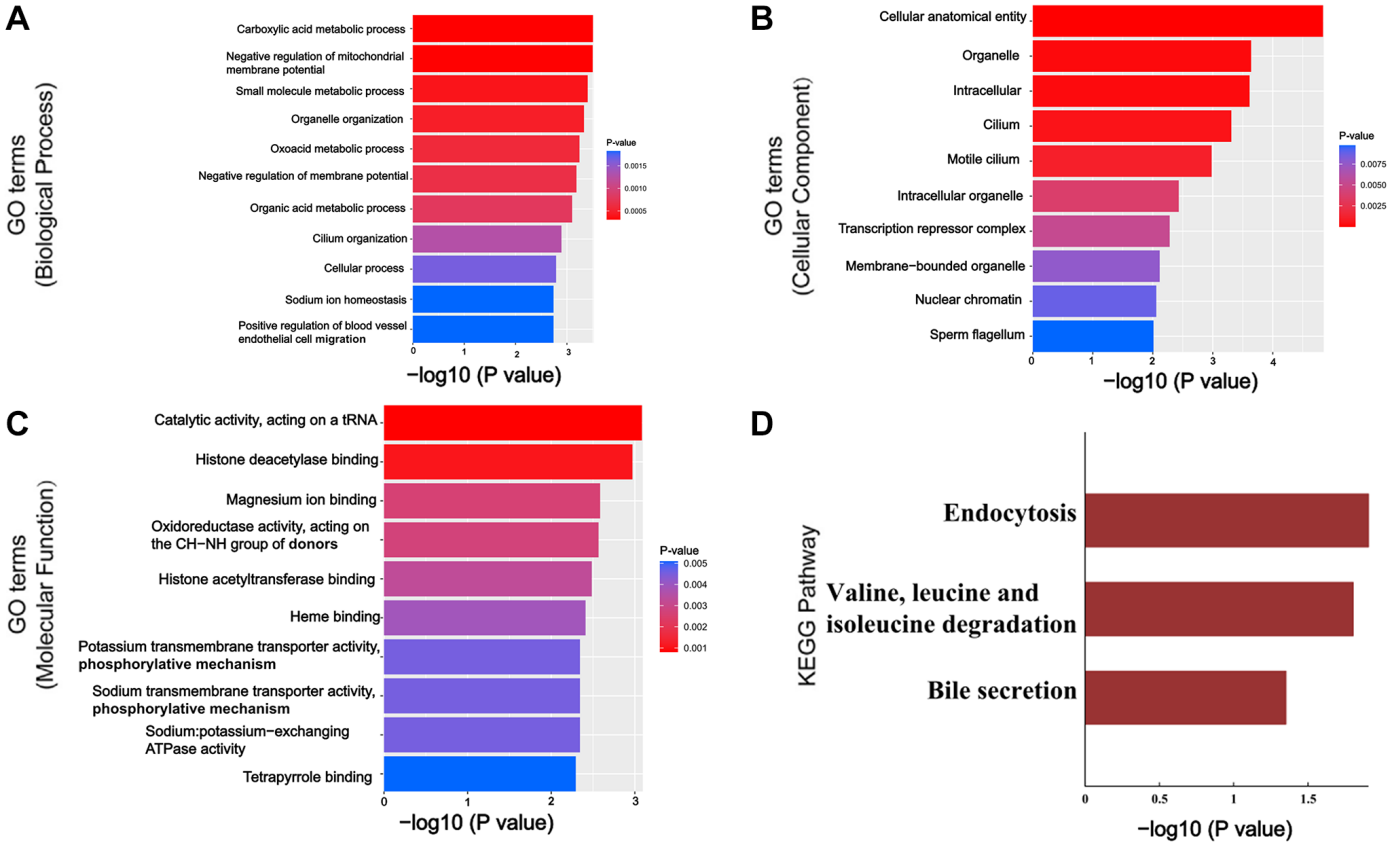
**GO and KEGG Pathway analysis of validated circRNAs related genes.** (**A**–**C**) Top 10 enriched GO terms in BP, CC and MF. (**D**) Top 3 enriched KEGG pathways. Abbreviations: GO: Gene Ontology; CC: cellular components; BP: biological processes; MF: molecular functions.

To explore the functional roles of circRNAs target genes, GO term enrichment and KEGG pathway analyses of these validated circRNAs related genes were performed. Specifically, the most significant GO functions were related to carboxylic acid metabolic process, negative regulation of mitochondrial membrane potential, small molecule metabolic process, cellular anatomical entity, organelle, intracellular, catalytic activity, acting on a tRNA, histone deacetylase and binding magnesium ion binding (Figure 6A–6C). Additionally, KEGG analysis identified numerous signaling pathways, associated with the dysregulated circRNAs-related genes, that were significantly altered in the OSA-induced liver injury. The KEGG pathways were significantly associated with Natural killer cell mediated cytotoxicity, one carbon pool by folate, Glycerolipid metabolism, HIF-1 signaling pathway, Renal cell carcinoma, cGMP-PKG signaling pathway, Central carbon metabolism in cancer, Thyroid hormone signaling pathway, Tuberculosis, β-Alanine metabolism (Figure 6D).

### Construction of a circRNA-miRNA-mRNA interaction network

**Figure 7:**
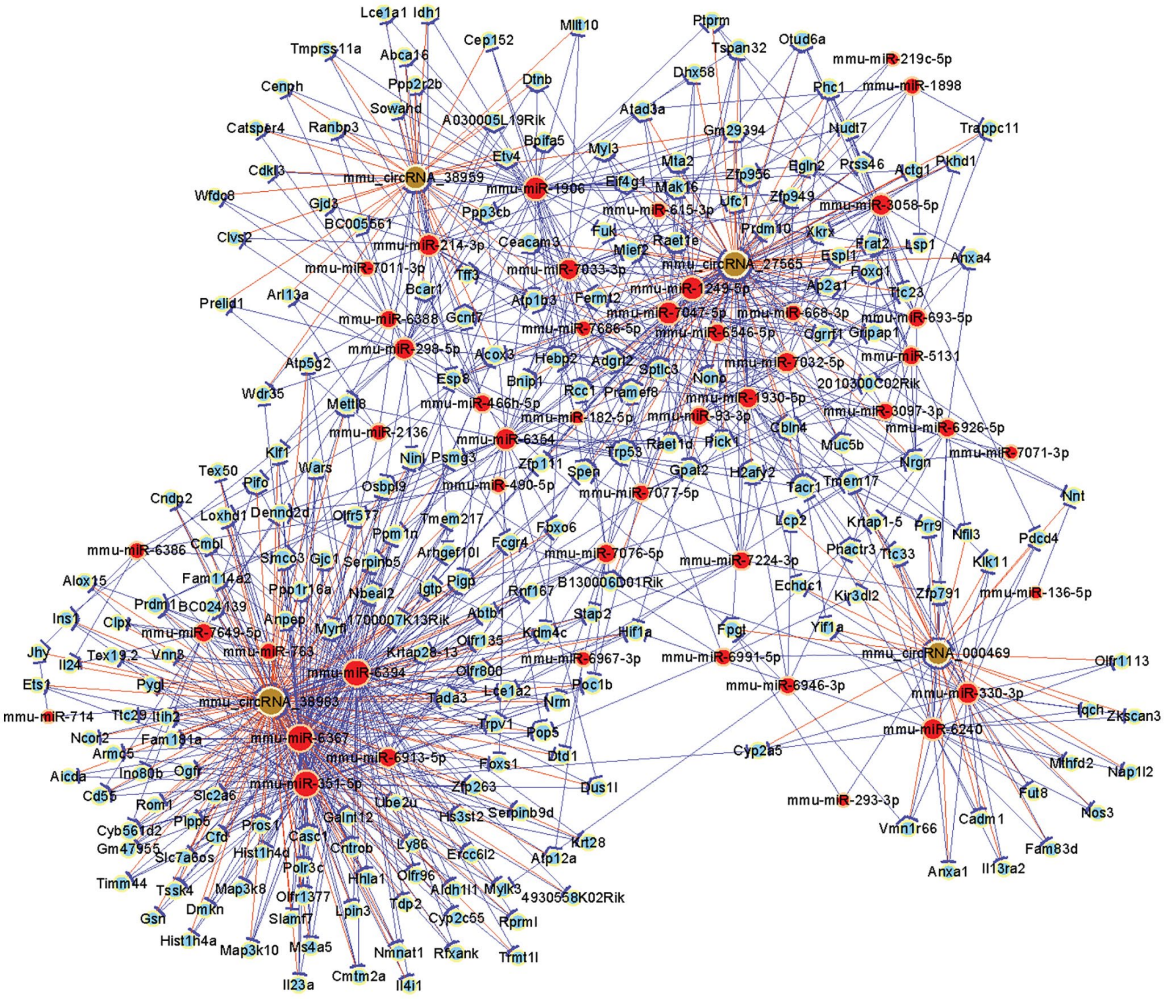
**A network diagram shows the circRNA-miRNA-mRNA network for four candidate circRNAs.** Yellow rounds, red rounds and green rounds represent circRNA, miRNA and mRNA, respectively.

For predictions of potential targets of the selected circRNAs, miRWalk, miRanda and Targetscan were used. To further explore the biological functions of circRNAs in OSA-induced liver injury, we constructed a lncRNA-miRNA-mRNA network, including four selected circRNAs (mmu_circRNA_000469, 38959, 38983, 27565), and their targeted miRNAs and downstream mRNAs. And the network was visualized by Cytoscape software (version 3.6.1) (Figure 7). There are 227 target miRNAs and corresponding 321 target mRNAs of the circRNAs by bioinformatics predictions. The interaction network will help us improve the understanding of underlying mechanism of mmu_circRNA_000469, 38959, 38983, 27565 by revealing its potential connections between circRNAs and miRNAs. Our finding suggested that these circRNAs may play a critical role in the biological process of OSA-induced liver injury.

## DISCUSSION

Accumulating studies indicate that OSA-related intermittent hypoxia may contribute to hepatic lipid accumulation by up-regulating a key hepatic transcription factor of lipid biosynthesis, sterol regulatory element binding protein 1c, via hypoxia-inducible factor 1 [16]. A linear relationship between the severity of sleep-related hypoxemia as assessed by the mean SaO2 and the risk of liver steatosis and cytolysis [17].

In this study, we performed a comprehensive analysis exploring the DECs in OSA-induced liver injury. And we conducted systemic bioinformatics analysis to identify some circRNAs essential for the biological processes of OSA-induced liver damage, which may provide potential targets for the development of novel diagnostic and therapeutic strategies against OSA-induced liver injury. With rapidly advancing research on the unique structural features and biological functions, circRNAs have become a focus of liver disease diagnosis, treatment, and have translational value in therapy [9].

In our study, compared to the control group, one circRNA is upregulated and 79 circRNAs are downregulated based on the high-throughput circRNA-sequencing data. Similarly, Other studies have shown that circRNA1056 and circRNA805, as two of DEGs, were predicted to interact with microRNAs in IH-induced BRL-3A cell injury [18]. However, mechanisms underlying the regulation of these 80 DEGs in OSA-induced liver injury remain unidentified.

In order to verify the reliability of high-throughput circRNA-sequencing data, six DECs (mmu_circRNA_000469, 37851, 38959, 38983, 31665, 27565) were randomly selected for validation. As expected, our RT-qPCR results of these six DECs are also consistent with the microarray, which gives us good confidence to do a stepwise bioinformatics analysis of DECs. GO analysis revealed that circRNAs-related genes were largely involved in processes associated with liver function such as carboxylic acid metabolic process and negative regulation of mitochondrial membrane potential, which may provide some useful information for understanding the biological functions of circRNAs-related genes in OSA-induced liver injury. Meanwhile, KEGG analysis found that there were 13 pathways related to these circRNAs-related genes, which were ranked in order of GeneRatio. And the most related pathway was Natural killer cell mediated cytotoxicity, which strongly suggests that immune responses may be of importance in the incidence and development of the OSA-induced liver injury. Activated NK cells release IFN-γ as well as the contents of cytotoxic granules, such as perforin and granzyme B, which contribute to hepatocellular necrosis [19].

The predicted ceRNA network can help to clarify the molecular mechanisms of dysregulated circRNAs in the OSA-induced liver injury. Interestingly, we revealed that mmu_circRNA_27565 can tightly bind to mmu-miR-1249-5p, which may be a potential sponge of mmu-miR-1249-5p. Meanwhile, Liying Li et al. reported that miR-1249-5p in negatively regulating macrophage MCP-1 expression during liver injury [20].

Moreover, we revealed that mmu_circRNA_27565 can target the protein interacting with C kinase 1 (PICK1). Jun Li et al. showed that PICK1 confers anti-inflammatory effects in acute liver injury in regulating macrophage polarization, implying PICK1 as a potential therapeutic target in ALI [21].

Our results are consistent with this view that PICK1 is a key molecule in the liver injury. Therefore, it is worth further study to determine the role of mmu_circRNA_27565 in the OSA-induced liver injury. However, due to the limited available data on the interaction of the circRNA_27565-miR-1249-5p-PICK1, future research should be specifically designed to investigate this ceRNA interaction. This study has a few limitations. The PCR validation of circRNAs was lack of divergent primers and gel electrophoresis, and the circRNAs also need further experimental validation and externally clinical cohort validation. In summary, our study identified the comprehensive expression profile of circRNAs in the OSA-induced liver injury. The circRNA-miRNA-mRNA interaction network prediction and bioinformatics analysis could provide a global understanding of mmu_circRNA_000469, 38959, 38983, 27565, which may be involved in the biological processes of OSA-induced liver injury. The comprehensive expression profile of circRNAs in the OSA-induced liver injury may contribute to the further study of potential diagnostic biomarkers or therapeutic targets.

## References

[1] Hong SN, Yun HC, Yoo JH, Lee SH. Association Between Hypercoagulability and Severe Obstructive Sleep Apnea. JAMA Otolaryngol Head Neck Surg. 2017; 143:996–1002. 10.1001/jamaoto.2017.1367 28817760 PMC5710255

[2] Porto F, Sakamoto YS, Salles C. Association between Obstructive Sleep Apnea and Myocardial Infarction: A Systematic Review. Arq Bras Cardiol. 2017; 108:361–9. 10.5935/abc.20170031 28380133 PMC5421476

[3] Lai S, Chen L, Zhan P, Lin G, Lin H, Huang H, Chen Q. Circular RNA Expression Profiles and Bioinformatic Analysis in Mouse Models of Obstructive Sleep Apnea-Induced Cardiac Injury: Novel Insights Into Pathogenesis. Front Cell Dev Biol. 2021; 9:767283. 10.3389/fcell.2021.767283 34820383 PMC8606653

[4] Tanné F, Gagnadoux F, Chazouillères O, Fleury B, Wendum D, Lasnier E, Lebeau B, Poupon R, Serfaty L. Chronic liver injury during obstructive sleep apnea. Hepatology. 2005; 41:1290–6. 10.1002/hep.20725 15915459

[5] Zhang L, Zhang X, Meng H, Li Y, Han T, Wang C. Obstructive sleep apnea and liver injury in severely obese patients with nonalcoholic fatty liver disease. Sleep Breath. 2020; 24:1515–21. 10.1007/s11325-020-02018-z 32002742

[6] Du WW, Yang W, Liu E, Yang Z, Dhaliwal P, Yang BB. Foxo3 circular RNA retards cell cycle progression via forming ternary complexes with p21 and CDK2. Nucleic Acids Res. 2016; 44:2846–58. 10.1093/nar/gkw027 26861625 PMC4824104

[7] Chen L, Kong R, Wu C, Wang S, Liu Z, Liu S, Li S, Chen T, Mao C, Liu S. Circ-MALAT1 Functions as Both an mRNA Translation Brake and a microRNA Sponge to Promote Self-Renewal of Hepatocellular Cancer Stem Cells. Adv Sci (Weinh). 2019; 7:1900949. 10.1002/advs.201900949 32099751 PMC7029649

[8] Fu L, Jiang Z, Li T, Hu Y, Guo J. Circular RNAs in hepatocellular carcinoma: Functions and implications. Cancer Med. 2018; 7:3101–9. 10.1002/cam4.1574 29856133 PMC6051148

[9] Fu LY, Wang SW, Hu MY, Jiang ZL, Shen LL, Zhou YP, Guo JM, Hu YR. Circular RNAs in liver diseases: Mechanisms and therapeutic targets. Life Sci. 2021; 264:118707. 10.1016/j.lfs.2020.118707 33144187

[10] Chen Q, Lin G, Huang J, Chen G, Huang X, Lin Q. Expression profile of long non-coding RNAs in rat models of OSA-induced cardiovascular disease: new insight into pathogenesis. Sleep Breath. 2019; 23:795–804. 10.1007/s11325-018-1753-0 30535531

[11] Ritchie ME, Phipson B, Wu D, Hu Y, Law CW, Shi W, Smyth GK. limma powers differential expression analyses for RNA-sequencing and microarray studies. Nucleic Acids Res. 2015; 43:e47. 10.1093/nar/gkv007 25605792 PMC4402510

[12] Dessau RB, Pipper CB. [''R"--project for statistical computing]. Ugeskr Laeger. 2008; 170:328–30. 18252159

[13] Wu T, Hu E, Xu S, Chen M, Guo P, Dai Z, Feng T, Zhou L, Tang W, Zhan L, Fu X, Liu S, Bo X, Yu G. clusterProfiler 4.0: A universal enrichment tool for interpreting omics data. Innovation (Camb). 2021; 2:100141. 10.1016/j.xinn.2021.100141 34557778 PMC8454663

[14] Yu G, Wang LG, Han Y, He QY. clusterProfiler: an R package for comparing biological themes among gene clusters. OMICS. 2012; 16:284–7. 10.1089/omi.2011.0118 22455463 PMC3339379

[15] Shannon P, Markiel A, Ozier O, Baliga NS, Wang JT, Ramage D, Amin N, Schwikowski B, Ideker T. Cytoscape: a software environment for integrated models of biomolecular interaction networks. Genome Res. 2003; 13:2498–504. 10.1101/gr.1239303 14597658 PMC403769

[16] Drager LF, Jun J, Polotsky VY. Obstructive sleep apnea and dyslipidemia: implications for atherosclerosis. Curr Opin Endocrinol Diabetes Obes. 2010; 17:161–5. 10.1097/MED.0b013e3283373624 20125003 PMC2904751

[17] Trzepizur W, Boursier J, Mansour Y, Le Vaillant M, Chollet S, Pigeanne T, Bizieux-Thaminy A, Humeau MP, Alizon C, Goupil F, Meslier N, Priou P, Calès P, Gagnadoux F, and Institut de Recherche en Santé Respiratoire des Pays de la Loire Sleep Cohort Group. Association Between Severity of Obstructive Sleep Apnea and Blood Markers of Liver Injury. Clin Gastroenterol Hepatol. 2016; 14:1657–61. 10.1016/j.cgh.2016.04.037 27155555

[18] Chen LD, Huang JF, Lin XJ, Huang YP, Xu QZ, Chen GP, Lin QC. Expression profiling and functional analysis of circular RNAs *in vitro* model of intermittent hypoxia-induced liver injury. Front Physiol. 2022; 13:972407. 10.3389/fphys.2022.972407 36187780 PMC9515621

[19] Dugan CM, Fullerton AM, Roth RA, Ganey PE. Natural killer cells mediate severe liver injury in a murine model of halothane hepatitis. Toxicol Sci. 2011; 120:507–18. 10.1093/toxsci/kfr005 21245496 PMC3061480

[20] Ji X, Yang L, Zhang Z, Zhang K, Chang N, Zhou X, Hou L, Yang L, Li L. Sphingosine 1-phosphate/microRNA-1249-5p/MCP-1 axis is involved in macrophage-associated inflammation in fatty liver injury in mice. Eur J Immunol. 2020; 50:1746–56. 10.1002/eji.201948351 32672363

[21] Xie J, Wu X, Zhou Q, Yang Y, Tian Y, Huang C, Meng X, Li J. PICK1 confers anti-inflammatory effects in acute liver injury via suppressing M1 macrophage polarization. Biochimie. 2016; 127:121–32. 10.1016/j.biochi.2016.05.002 27157267

